# Effects of Postprandial Factors and Second Meal Intake Time on Bioequivalence Investigation of Tadalafil-Loaded Orodispersible Films in Human Volunteers

**DOI:** 10.3390/pharmaceutics16070915

**Published:** 2024-07-09

**Authors:** Su-Jun Park, Myung-Chul Gil, Bong-Sang Lee, Minji Jung, Beom-Jin Lee

**Affiliations:** 1 Department of Pharmacy, College of Pharmacy, Ajou University, Suwon-si 16499, Republic of Korea; klevel2@hanmail.net; 2 CTCBIO Inc., Hwaseong-si 18576, Republic of Korea; 3 PLUTO Inc., Seongnam-si 13453, Republic of Korea; mcgil@pluto5.co.kr (M.-C.G.); keberos@pluto5.co.kr (B.-S.L.); 4 Department of Urology, School of Medicine, Stanford University, Stanford, CA 94305, USA; mjjung@stanford.edu; 5 Institute of Pharmaceutical Science and Technology, Ajou University, Suwon-si 16499, Republic of Korea

**Keywords:** orodispersible film, tadalafil, drug particle size, in vitro dissolution, postprandial condition, meal viscosity, second meal intake time, in vivo bioequivalence, computational fluid dynamics

## Abstract

Tadalafil (TD) has poor water solubility but is well absorbed without affecting food intake when administered orally. Owing to patient adherence and therapeutic characteristics, a TD-loaded orodispersible film (TDF) is preferable. However, the mechanistic role of dietary status on the clinical pharmacokinetic analysis of TDF in human volunteers should be investigated because the gastrointestinal environment varies periodically according to meal intervals, although commercial 20 mg TD-loaded tablets (TD-TAB, Cialis^®^ tablet) may be taken with or without food. TDF was prepared by dispersing TD in an aqueous solution and polyethylene glycol 400 to ensure good dispersibility of the TD particles. In the fasting state, each T/R of Cmax and AUC between TD-TAB and TDF showed bioequivalence with 0.936–1.105 and 1.012–1.153, respectively, and dissolution rates in 1000 mL water containing 0.5% SLS were equivalent. In contrast, TDF was not bioequivalent to TD-TAB under the fed conditions by the C_max_ T/R of 0.610–0.798. The increased dissolution rate of TDF via the micronization of drug particles and the reduced viscosity of the second meal content did not significantly affect the bioequivalence. Interestingly, an increase in second meal intake time from 4 h to 6 h resulted in the bioequivalence by the Cmax T/R of 0.851–0.998 of TD-TAB and TDF. The predictive diffusion direction model for physical digestion of TD-TAB and TDF in the stomach after the first and second meal intake was successfully simulated using computational fluid dynamics modeling, accounting for the delayed drug diffusion of TDF caused by prolonged digestion of stomach contents under postprandial conditions.

## 1. Introduction

Tadalafil (TD) is a selective cyclic guanosine monophosphate (cGMP)-specific phosphodiesterase type 5 inhibitor used to treat erectile dysfunction (Figure S1). It is a poorly water-soluble drug [Biopharmaceutics Classification System (BCS) class II] with a molecular weight of 389.41 and a high absorption rate. Its bioavailability is highly affected by the particle size of the drugs [1,2], but not food [3,4,5,6]. However, the absolute bioavailability of tadalafil following oral dosing has not been determined in any clinical study [3]. Commercially available oral TD dosage forms at daily doses of 20, 10, 5, and 2.5 mg are administered with or without food intake [7]. To enhance patient compliance, orodispersible film (ODF) may also be administered [8]. Despite being classified as a solid dosage form, ODF has distinct physical properties and undergoes unique manufacturing processes compared to other solid dosage forms such as tablets [9,10,11,12,13,14,15].

Various methods have been used for preparing ODF formulations with enhanced therapeutic efficiency through oral mucosal absorption [16,17,18,19,20,21]. Many studies have focused on effectively utilizing ODF formulations to improve the bioavailability or bioequivalence of drugs by changing the mucosal administration route to bypass the hepatic first-pass effect or encapsulating micronized drugs to improve the water solubility [16,22]. Various ODF formulations have been developed and their pharmacokinetics and bioequivalence have been compared with those of reference solid dosage forms [23,24,25].

However, the in vitro and in vivo behaviors of ODF formulations cannot be predicted from those of the solid dosage forms [26,27]. For example, ODF is administered after almost complete disintegration in the oral cavity, unlike solid dosage forms, which disintegrate gradually [28]. Rapid drug release followed by the rapid onset of efficacy is one of the pharmaceutical advantages of ODF [9,11,28]. The bioequivalence of various dosage forms is commonly studied under fasting or fed conditions [23,25]. Previous studies investigating the in vitro/in vivo correlation (IVIVC) of immediate-release and orally dissolving tablets in the stomach after meals have reported that gastric viscosity and delay in gastric emptying time (GET) are correlated [26,27,28]. However, the postprandial pharmacokinetic profiles of immediate-release and orally dissolving tablets may differ. The effect of delaying the GET of dissolvable tablets may be minimal because of the food effect in the postprandial state. In particular, considering the calories and viscosity of food and their effect on GET is not only relevant to the high-fat diet but also addresses the influence of large food particles in the stomach, which can lead to short mealtimes of approximately 15 min [29]. Since the physical behavior of food in the stomach significantly varies depending on its composition and content, the formulation types administered and the post-meal stomach environment should also be considered. The critical physicochemical factors, pharmacokinetics, and bioavailability of various model drugs in orally dispersible formulations and conventional immediate-release dosage forms were compared, indicating the irrelevance of water intake and biopharmaceutical performance [30]. In a previous study on the influence of food effect of drugs [27], the feeding effect does not appear in drugs administered in the form of solutions and/or soluble tablets. In a study of TD-loaded ODF, no direct evidence for the pre-gastric absorption effect of ODF and the pharmacokinetic profile of ODF formulation under fed conditions has been described [31]. In another study conducted under fasting conditions, the pharmacokinetic bioequivalence between TD-loaded ODF and tablets was reported but no further evidence for bioequivalence under fed conditions was provided [32]. 

In addition, CFD models have been utilized for investigating the flow dynamics of liquid gastric contents in the human stomach induced by gastric motility. Among the post-meal gastrointestinal movement models [33,34,35,36,37], the CFD model offers a relatively comprehensive approach for inferring the complex postprandial aspects of gastrointestinal movement and explains the effect of GET based on the actual physical behavior of the stomach contents [33,34]. Although several studies have explored the flow in the stomach [35], they have often overlooked the mechanical aspects of gastric movement [38,39,40,41] or have been conducted under specific conditions using specialized equipment [35,40,42,43,44], limiting the generalizability of the results. In addition, previous studies have not clearly elucidated drug behavior based on the dosage form under fed conditions, reflecting realistic dietary conditions [45]. The CFD model appears to be appropriate for understanding the behavior of food content in the stomach after meal intake. Therefore, understanding the fluid dynamics of gastric digestion using CFD modeling should be very useful to investigate gastrointestinal behaviors of various dosage forms, resulting in bioavailability. 

However, the effects of meal intake time and postprandial conditions on the pharmacokinetics and bioavailability of drug-loaded ODF formulations are controversial and have rarely been investigated. Furthermore, there are not enough pharmacokinetics and bioequivalence cases of TD-loaded ODF under fed conditions in human subjects. For these reasons, the current study may provide a new approach to developing and predicting ODF.

This study aimed to design TD-loaded ODF (TDF) formulations and compare their bioequivalence with commercial 20 mg TD-loaded tablets (TD-TAB, Cialis^®^ tablet) in healthy human volunteers. The effects of drug particle size, meal content viscosity, and second meal intake time on the bioequivalence of TDF and TD-TAB were evaluated. Finally, the predictive diffusion direction and drug release by digestion and gastric emptying of TD-TAB and TDF in the stomach after the first and second meal intake, leading to the difference in pharmacokinetic profiles, were successfully suggested using the concept of computational fluid dynamics (CFD) modeling.

## 2. Materials and Methods

### 2.1. Materials

Materials and reagents for sample preparation and formulation researches were purchased as follows: TD (Purity ≥ 99.0%, Mylan, Hyderabad, India), hydroxypropylmethylcellulose (HPMC, Methocel™ E5 Premuim LV, IFF, Freienbach, Switzerland), hydroxypropylcellulose (HPC-SL, Nisso HPC, Joetsu-shi, Japan), pullulan (Hayashibara, Okayama, Japan), polyethylene glycol 400 (PEG 400, Carbowax™, Dow Chemical, La Mirada, CA, USA), polyoxyl 35 castor oil (Kolliphor EL^®^, BASF, Ludwigshafen, Germany), polyethylene glycol 40 hydrogenated castor oil (Kolliphor^®^ RH40, BASF, Ludwigshafen, Germany), triethyl citrate (Merck, Burlington, VT, USA), polyethylene oxide (N80, Polyox^TM^, IFF, Oegstgeest, the Netherlands), polyvinyl alcohol–polyethylene glycol copolymer (Kollicoat^®^ IR, BASF, Ludwigshafen, Germany), sodium lauryl sulfate (Nacalai, Kyoto, Japan), trifluoroacetic acid (Sigma-Aldrich, St. Louis, MO, USA). Sodium chloride, potassium phosphate, phosphoric acid, and sodium hydroxide were obtained from Merck (Darmstadt, Germany). High-performance liquid chromatography (HPLC)-grade acetonitrile and methanol were purchased from Honeywell (Muskegon, MI, USA). Purified water was obtained in-house (Arium^®^ Pro, Satorius, Göttingen, Germany). The reference drug (TD-TAB; Cialis^®^ tablet 20 mg, Lilly del Caribe Inc., Carolina, Puerto Rico) was used in the comparative dissolution and pharmacokinetic study.

### 2.2. Preliminary Screening of Dispersion Agents

TD is a BCS class II drug whose particle size significantly impacts solubility and bioavailability [2]. Although previous studies have attempted to increase the low solubility (3 μg/mL) of TD to improve its bioavailability, the in vitro and in vivo correlation (IVIVC) related to increased solubility in ODF formulations has not been verified [46,47]. To stably disperse undissolved micronized TD within the ODF matrix, approximately 5% dispersing agent was dispersed to each candidate polymer (5%) and dried at room temperature (25 °C). The polymers chosen were HPMC (5cp), HPC-SL, and pullulan. The dispersion agents selected were PEG 400, polyoxyl 35 castor oil, polyethylene glycol 40 hydrogenated castor oil, triethyl citrate, polyethylene oxide, and polyvinylalcohol–polyethylene glycol copolymer. The visual appearance, possible TD precipitation, and content uniformity, expressed as the relative standard deviation (RSD) % of the film, were characterized to confirm good dispersibility. The TDF (200 × 400 mm^2^) was collected and cut into 30 films (37 × 27 mm^2^). Then, 10 sheets were taken, and the content was measured. A combined solution of the dispersing agent and polymers with the lowest % RSD without any cracks, TD precipitation, or non-homogeneity was selected. The film solution containing the dispersing agent and the application and drying methods were used as a TDF formulation.

### 2.3. TDF Formulation Preparation

Based on the preliminary screening of dispersion agents into polymers, uniformly distributed optimal TDFs were prepared as follows: 17.01% TD, 51.92% hydroxypropylcellulose, 0.17% xanthan gum, 2.55% polyethylene glycol 400, 11.06% glycerin, 0.85% polysorbate 80, diluents, colorants, 17.31% sweeteners; the total weight of 1 film was 117.55 mg. This combination was added to purified water and homogenized for 30 min at 3000 rpm using a homogenizer (T-25; IKA, Königswinter, Germany). The film solution was degassed under vacuum and applied to PET film to a thickness of approximately 400 μm and a width of approximately 150 mm. TDF was prepared by drying in a drying oven at 70 °C for 40 min and peeling from the PET film.

To investigate the effect of particle size of TD on bioequivalence, two TDFs containing 12.4 or 7.8 μm TD were manufactured based on the particle size distribution (PSD) D90 of TD, and then designated as TDF-1 (D90: 12.4 μm) and TDF-2 (D90: 7.8 μm). The final prepared TDFs had a size of 37 × 27 mm^2^ based on the 20 mg TD dose.

### 2.4. Physicochemical Characterization of TDF Formulations

#### 2.4.1. Scanning Electron Microscopy (SEM)

The TD particles were analyzed using a scanning electron microscope (JEOL, Akishima, Japan), targeting the TD powder and TD contained in the ODF. Briefly, 3–5 mg powder or ODF was taken, and their surface images were captured at 3–5 kV acceleration voltage.

#### 2.4.2. Particle Size Distribution (PSD)

The particle size distribution (PSD) was measured using a PSD analyzer (Mastersizer 3000, Malvern, UK) according to the wet method. TD was added to water containing 2% polyethylene glycol 400, the strongly stirred solution was sonicated, and the PSD measurement equipment was used. The samples were measured by dropwise addition to the dispersion unit and stirring at 3000–3500 rpm. Sonication power was adjusted to 50–80%.

#### 2.4.3. Dissolution Study

The comparative dissolution test of TD-TAB and TDF was conducted according to <711> Dissolution, general chapter, USP, using the apparatus 2 paddle method at 50 rpm and 37.5 ± 5 °C in 1000 mL water containing 0.5% SLS, according to the “Dissolution method database, FDA”. TDF was rolled into a round shape, placed in a sinker, and used in an automatic dissolution device (VK7000; VARIAN, Cary, NC, USA). Based on the dissolution data, the similarity factor (*f*_2_), which measures the closeness between two dissolution profiles, was calculated according to the equation below, where *n* is the number of time points, and *R_t_* and *T_t_* are the dissolution values of TD-TAB and TDFs at time *t*, respectively.

$$f_{2}=50\times \log\left\{{\left[1+\left(1/n\right)\sum_{t=1}^{n}{\left(R_{t}-T_{t}\right)}^{2}\right]}^{-0.5}\times 100\right\}$$

#### 2.4.4. Disintegration Test

The disintegration tests of TDFs were conducted according to “<701> Disintegration, general chapter, USP” with disks. Each film was put in each tube of Basket-rack assembly of disintegration apparatus in water at 37 ± 2 °C and 29–32 cycle/min. The disintegration completion point was determined based on the time at which all films were completely invisible in the tube of the disintegration tester (DIT-200, Labfine INC., Gunpo-si, Republic of Korea).

### 2.5. Analysis of TD Uniformity in TDF

The % RSD to confirm the drug uniformity and dissolution profiles of TDFs was analyzed using an HPLC system with an octylsilyl silica column (5 μm, 250 mm) and 50 μL injection volume. The mobile phase used was a 55:45 mixture of PBS solution containing 0.1% (*v*/*v*) trifluoroacetic acid (TFA) and acetonitrile. The flow rate of the mobile phase was 1.2 mL/min. The column temperature was 40 °C. Through validation of the analytical method, the standard calibration was validated for the linearity of R^2^ ≥ 0.999 and precision of RSD ≤ 1.0% at 0.0025–0.02 mg/mL TD.

### 2.6. The Simulation of Meal Viscosity by Water Intake Volumes

To simulate the relative viscosity of meal composition under postprandial stomach conditions, the fat-simulated meal compositions (481 g) were dispersed in three different solutions: 50 mL sodium phosphate (pH 6.8), 100 mL 0.01 N HCl, and 20, 150, or 240 mL of tap water containing 200 mL whole milk (total volume: 370, 500, or 590 mL, respectively) in a 1000 mL beaker. The solution viscosity was measured every 5 min for 30 min using a rotational viscometer (RVDV2T viscometer, Brook field, Middleboro, MA, USA) with a No. 2 spindle.

### 2.7. The Drug Diffusion Rate in the Postprandial Stomach Conditions

To simulate the drug diffusion rate from dosage forms in the postprandial stomach, the fat-simulated meal was ground for 30 s using a hand-blender (HR1613/00, Phillips, Andover, MA, USA), and 50 mL sodium phosphate was added to a dissolution tester (VK7000, VARIAN, Cary, NC, USA) vessel. The temperature of the meal contents was adjusted to 37 ± 5 °C (50 rpm) and 100 mL 0.01 N HCl was added to each dissolution vessel. Approximately 30 min later, TD-TAB was inserted into a vessel without disintegration by dropping the tablet in 150 mL water, while TDF was dissolved in 20 mL water and inserted in a vessel. After dissolution, the paddle was rotated at 50 rpm, and a 2 mL sample was collected from the lower layers of the TD-TAB or TDF vessels for 60 min. Each sampled solution was centrifuged, and the supernatant was filtered again through a 0.45 μm filter for further studies. The TD-TAB and TDF loading concentrations were 20 mg/500 mL and 20 mg/370 mL, respectively. The TD concentrations in the samples were analyzed using HPLC as in the dissolution analysis method. The TD concentrations in the lower part of the dissolution vessel were plotted as a function of time to simulate the diffusion directions of 20 mg TD-TAB and 20 mg TDF-1.

### 2.8. Pharmacokinetics and Bioequivalence Test in Healthy Human Volunteers

#### 2.8.1. Design and Dosing Schedule

The protocol (CDFF0213-01) of the pharmacokinetic study was approved by the Institutional Review Board (YJCTC_IRB_011, offline documentation) of Yangji Hospital (Seoul, Republic of Korea) and was conducted in accordance with the Declaration of Helsinki (October 1996) for biomedical research involving human subjects and the International Conference on Harmonisation (ICH) E6 Guideline for Good Clinical Practice (GCP). A detailed explanation of the study was provided to each participant, and their written informed consents were obtained prior to screening. This phase 1 clinical study was also submitted to and approved by the Ministry of Food and Drug Safety, The Republic of Korea (Reception number: 20130111986, 4 July 2013; approved protocol: CDFF0213-01).

The dosing schedule of ODFs for investigating the pharmacokinetics and bioequivalence in healthy human volunteers is shown in Table 1. The dosing schedules of the ODF formulations varied according to the formulation, water volume, and meal conditions.

#### 2.8.2. Fasting-State Study

A fasting condition pharmacokinetic study of TDF-1 was conducted in a randomized, single dose, two-way crossover, open-design method targeting 40 healthy men aged 20–55 years, using the original drug Cialis^®^ tablet as the reference group. The participants ate the same dinner the day before oral administration, fasted until 8 a.m. the next day, and were administered each group of drugs according to the medication order and procedure. Water intake was restricted from 1 h before to 2 h after drug administration. Lunch and dinner were provided as standard meals 4 and 10 h after administration, respectively [48,49,50].

Blood was collected 16 times at 0, 0.33, 0.67, 1, 1.5, 2, 2.5, 3, 4, 6, 8, 12, 24, 48, 72, and 96 h after administration [49,50]. After a 14-d drug-free washout period, the second phase of the crossover test between TD-TAB and TDF was performed using the same procedure. The TD-TAB group took a 20 mg Cialis^®^ tablet with 240 mL water. The TDF group wetted their mouth with 20 mL water, and then TDF was placed on the tongue for approximately 1 min to completely dissolve it before swallowing it with saliva.

#### 2.8.3. Fed-State Study

The fed condition pharmacokinetic study of TDF was conducted in a randomized, single dose, 2-way crossover, open design targeting 8–12 healthy men aged 20–55 years, using the original drug Cialis^®^ as the reference group. The subjects ate the same dinner the day before administration, remained fasted until 7 a.m. the next day, and completed breakfast within 30 min [45]. The meal compositions were set as a high-fat diet in accordance with the recommended calorie and nutrient ratios in the postprandial pharmacokinetic test, according to FDA guidance. Fat-simulated meal compositions (total 481 g) consisted of double cheeseburger 1, fried potato (regular size), and 200 mL of whole milk. The total calories were 900–1000 kcal, giving about 150 kcal (15%) from protein, about 250 kcal (25%) from carbohydrates, and about 500–600 kcal (50–60%) from fat [45]. After providing the breakfast meal, the dosage forms (TD-TAB, TDF-1, or TDF-2) were administered within 30 min according to the same procedures in the fasting-state study. Depending on the water volume (20 or 240 mL) and second meal intake time, the dosing studies were designated as Fed-I, II, III, and IV, respectively (see Table 1). Blood was collected at 0.33, 0.67, 1, 1.5, 2, 2.5, 3, 4, 5, 6, 8, 12, 24, 33, and 48 h after administration [49,50]. 

#### 2.8.4. Analysis of Drug Concentration in Blood

The TD concentration in plasma was analyzed in the validated concentration range of 2–1000 ng/mL using liquid chromatography-tandem mass spectrometry (LC-MS/MS). TD concentration was determined from a previously prepared calibration curve by calculating the ratio of the TD peak area to the internal standard peak area in the analyzed chromatogram. The analytical method was validated through specificity, carry-over, matrix effect, recovery, calibration curve, accuracy, precision, stability, and dilution effects, which were suitable.

Standard TD was dissolved in methanol to 1 mg/mL, stored frozen, and diluted with frozen blank plasma to determine the plasma concentration of TD at 2, 5, 10, 50, 100, 500, and 1000 ng/mL. Plasma samples were prepared in milliliters. Briefly, 50 μL internal standard (10 ng/mL buflomedil in 50% acetonitrile) was added to 50 μL standard plasma and mixed. Then, 400 μL acetonitrile was added, mixed with a vortex mixer for 10 s, and centrifuged at 12,000 rpm (18,514× *g*) for 5 min. Finally, 5 μL supernatant was injected into the LC-MS/MS system, and a calibration curve was created by calculating the ratio of the TD peak area to the internal standard peak area. Each plasma sample collected from a participant and stored at −70 °C or lower was thawed at room temperature, dissolved, and shaken. Then, 50 μL of this plasma was pretreated in the same manner as the calibration curve preparation method and injected into the LC-MS/MS system. For the analysis, a column packed with octadecylsilyl silica gel (Hipersil Gold^TM^ C18 selectivity 100 mm × 2.1 mm, 1.9 μm, Thermo Fisher Scientific^TM^, Waltham, MA, USA) was used. The sample injected at 5 μL was analyzed at a flow rate of 0.3 mL/min. using a mobile phase containing 0.1% formic acid and acetonitrile in a ratio of 20:80, and the detector (Thermo Finnigan TSQ Vantage, Thermo Fisher Scientific^TM^, Waltham, MA, USA) was used in MRM (Multiple Reaction Monitoring) mode.

#### 2.8.5. Pharmacokinetic Parameter Calculation and Analysis

The pharmacokinetic parameters were determined directly from the plasma concentration of TD over time using the Phoenix™ WinNonlin^®^ (Pharsight, CA, USA) program. The 90% confidence interval (CI) of the log-transformed mean difference of area under the curve (AUC), the maximum TD concentration (C_max_), and the time to reach C_max_ (T_max_) were calculated for verifying the bioequivalence of TD-TAB and TDF formulations. For AUC_t_ and C_max_, the point estimate and range of the ratio of the geometric mean of the reference and test drugs were considered bioequivalent of AUC and C_max_, ranging from log 0.8 to log 1.25 at the 90% CI. T_max_ was used for comparison.

## 3. Results and Discussion

### 3.1. Screening of Dispersion Agents and Formulation Design

Table 2 presents the visual appearance and % RSD of the polymers with the dispersion agents. Among the dispersing agents used to prepare TDF, PEG 400 generally showed the best results when combined with HPMC, HPC-SL, and pullulan, with 0.44, 0.43, and 0.31 relative (%) RSD, respectively. Although the enhanced solubility of TD-based dosage forms is important for improving oral bioavailability, PEG 400 did not significantly increase the solubility of TD, but properly dispersed TD in an aqueous solution, making it a highly desirable dispersing agent [21]. Based on the visual appearance, which had no drug precipitation, surface cracks or roughness, or non-homogeneity, the combination of HPC-SL and PEG400 was finally selected for further formulation studies.

**Figure 1:**
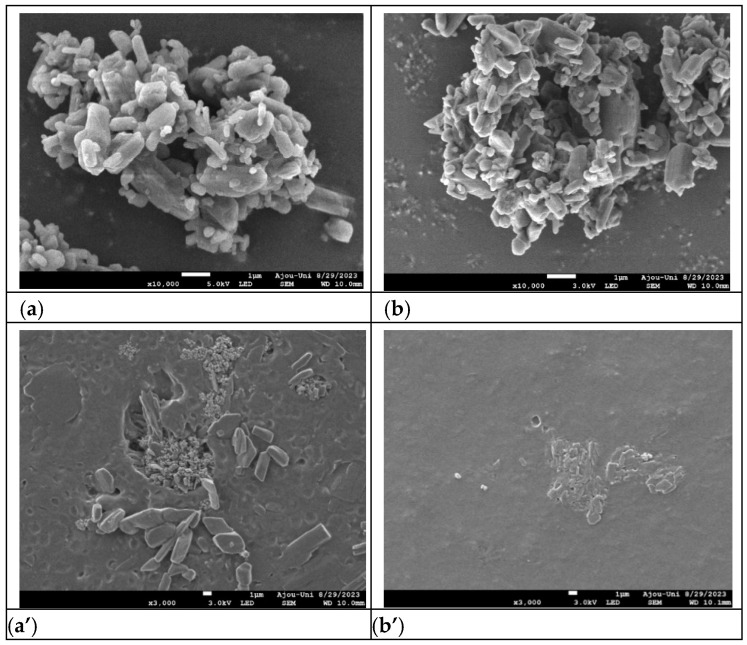
The SEM morphology with different tadalafil (TD) particle sizes (**top**) and surfaces of TD-filled orodispersible films (TDFs; **bottom**). (**a**) TDF-1 TD particles (10,000×), (**a′**) TDF-1 surface (3000×), (**b**) TDF-2 TD particles (10,000×), (**b′**) TDF-2 surface (3000×).

The D90 of TD in TDF-1 and TDF-2 was 12.4 and 7.8 μm, respectively (Figure S2). Figure 1 shows the SEM morphology with different TD particle sizes and surfaces of TDF-1 and TDF-2. TDF-2, which dispersed more micronized TD particles, showed a relatively dense crystal form compared to TDF-1. The effect of TD particle size significantly influenced drug bioavailability [1,2,51].

### 3.2. The Disintegration and Dissolution Rate of TD-Loaded Formulations

**Figure 2:**
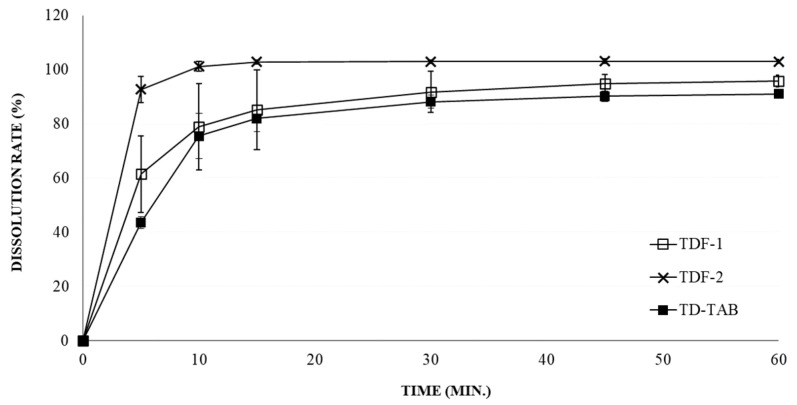
The dissolution rate of tadalafil (TD)-loaded formulations in water containing 0.5% SLS.

Figure 2 shows comparative dissolution rates of TD-loaded formulations in water containing 0.5% SLS. The overall dissolution rate of TDF-2 was much higher than that of TDF-1 and TD-TAB. The initial dissolution rates of TDF-1 and TDF-2 were significantly different, being 62% and 92% after 5 min, respectively. The f2 was calculated from the dissolution data [52], which was significantly different, between TDF-1 and TDF-2. The f2 for TDF-1 and TD-TAB was 77.04, indicating high similarity, whereas that for TFD-2 and TD-TAB was 33.95 (<50), suggesting high non-similarity. The dissolution rate was independent of the disintegration time of TDF formulations, which was 40.8 ± 3.76 and 40.8 ± 5.84 s for TDF-1 and TDF-2, respectively.

### 3.3. The Drug Diffusion Rate in the Postprandial Stomach Conditions

**Figure 3:**
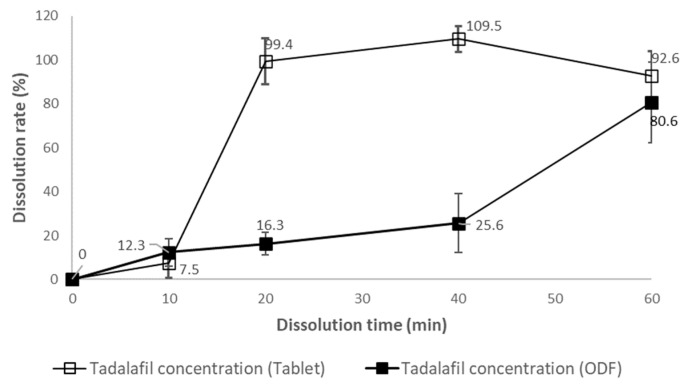
The diffusional rate of TD concentrations at the lower region of the dissolution vessel under the fed-simulated conditions to elucidate the diffusional direction of 20 mg TD-TAB (Cialis^®^) and 20 mg TDF-1.

Figure 3 gives the drug diffusional rate of TD concentrations at the lower part of the dissolution vessel under the fed-simulated conditions to elucidate the diffusion direction of 20 mg TD-TAB (Cialis^®^) and 20 mg TDF-1. TD-TAB almost reached peak dissolution in the lower region within 20 min of release initiation and maintained high dissolution until 60 min. However, approximately 60 min or more was required to release the drug until the TD diffused from the surface to the lower region of the dissolution vessel. The TD concentration from TD-TAB for 40 min was substantially higher than that from TFD-1 at the lower part of the vessel in fed-simulated conditions, suggesting that TD-TAB dispersion and dissolution occurred in the lower region of the stomach, unlike the TDF formulation. In addition, the time required for concentration gradient and digestive homogenization by gastric contraction at the diffusion location of dosage forms in the postprandial state is also important.

### 3.4. Comparative Bioequivalence Studies

**Figure 4:**
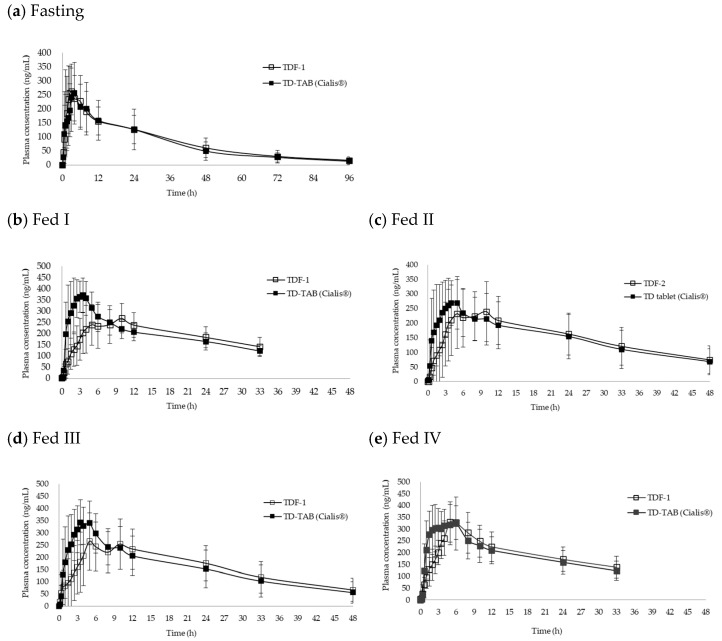
The comparative pharmacokinetic profiles of TDF and TD-TAB (Cialis^®^) in healthy human volunteers under fasting and fed states. (**a**) Fasting, (**b**) Fed I, (**c**) Fed II, (**d**) Fed III, (**e**) Fed IV.

The comparative pharmacokinetic profiles of TDF and TD-TAB in healthy human volunteers in the fasting and fed states are shown in Figure 4. Table 3 also compares the pharmacokinetic parameters of TDF and references TD-TAB (Cialis^®^) in healthy human volunteers under fasting and fed states. In the fasting state, the C_max_ and AUC (90% CI) of TDF-1 were 0.936–1.105 and 1.012–1.153, respectively, showing bioequivalence with TD-TAB; the T_max_ of TDF-1 and TD-TAB (Cialis^®^) were also similar (3.2 ± 2.2 and 2.6 ± 1.7 h, respectively; Figure 4a). The high similarity in the comparative dissolution profiles (Figure 2) suggests that TDF-1 was substituted with TD-TAB in the fasting state.

In contrast, clinical pharmacokinetics and bioequivalence were highly variable, depending on parameters such as TDF type, viscosity of meal contents, and second meal intake time. In the same experiment, except for first and second meal intake conditions, the plasma concentration of TD-TAB rapidly increased, reaching a peak within 2 or 3 h. In contrast, plasma concentrations of TDF-1 and TDF-2 increased slowly. Furthermore, the Fed I, II, and III conditions exhibited a double-peak phenomenon, except for the Fed IV condition.

Under the Fed I condition, the T/R ratio (90% CI) of C_max_ and AUC between TDF-1 and TD-TAB are 0.610–0.798 and 0.884–1.022, respectively, and the C_max_ was lower than that of TD-TAB, showing non-bioequivalence (Figure 4b). Interestingly, the T_max_ of TDF-1 was approximately three times slower than that of TD-TAB, but the observed AUC, reflecting overall drug exposure in the body [53], was reasonably within the bioequivalence ranges between the two dosage forms. Although oral TD-TAB was diet-independent [3,4,5,6], meal intake significantly affected the bioequivalence of TDF, mainly C_max_. The lower C_max_ and delayed T_max_ compared to TD-TAB suggested inherent unique formulation characteristics of ODF [16,24,25,54,55]. Based on the bioequivalent ranges of AUC, these effects were due to dilution of the TD dosage form and delayed GET, rather than the inhibition of drug absorption [27].

The bioavailability of TD is significantly influenced by particle size [1,2,51]. The bioequivalence was also studied under the same fed state, except for TDF-2, containing smaller TD particle sizes (Fed II). 

The T/R of AUC between TD-TAB and TDF-2 (0.894−1.137) was within the bioequivalence ranges, but the C_max_ was also low (0.735−0.972), showing non-bioequivalence (Figure 4c). This was similar to that of TDF-1, regardless of the decreased TD particle size in TDF-2, which had a faster and higher dissolution rate than TD-TAB. It was predicted that the reduction in the TD particle size further increased C_max_. However, it is crucial to note the simultaneous increase in the AUC. Previous studies have explored the enhancement of bioavailability by decreasing the particle size of poorly soluble drugs, indicating a strong association between particle size reduction and increased bioavailability [1,20,56]. Considering that AUC is one of the critical factors that reflects the amount of drug entering systemic circulation, in terms of bioavailability [53], TDF-2 showed a >10% increase in AUC compared to TD-TAB, suggesting that further particle size reduction could increase bioavailability. However, the reduction in TD particle size in TDF formulations was more sensitive to postprandial conditions, unexpectedly decreasing the C_max_ compared to that in the Fed I condition. This suggests that the variations in the two main parameters (C_max_ and AUC) by reducing the TD particle size indicate non-bioequivalence under postprandial conditions between the TD-TAB and TDF formulations.

Unlike the TD-TAB, the TDF formulation requires little or no water for oral administration for better patient dosing convenience [54,55]. However, differences in water intake volumes during administration, particularly in postprandial conditions, may alter the viscosity of meal contents and drug dissolution rates in the stomach environment, as supported by the CFD model, which offers a relatively comprehensive approach to inferring the postprandial complex aspects of gastrointestinal movement [33,34,35,36,57]. It showed that the discharge of gastric contents was closely related to viscosity [26,27].

**Figure 5:**
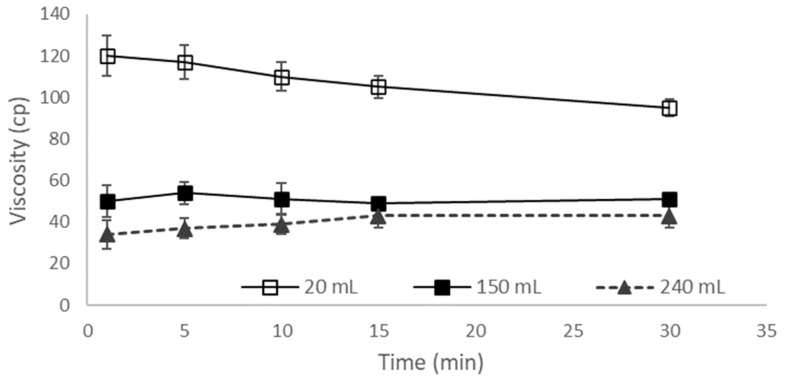
Relative viscosity to simulate postprandial conditions of TD-TAB (150 or 240 mL) and TDF-1 (20 mL) in the stomach according to three different water intake volumes.

Water (240 or 150 mL) is consumed when administering oral tablets in the postprandial state, but 20 mL water is consumed when ODF formulations are administered according to FDA guidelines [45,48]. Figure 5 shows the relative viscosity variations used to simulate postprandial conditions in the stomach according to the three different water intake volumes. Consuming 150 and 240 mL of water showed similar relative viscosities. The relative gastric viscosity after consuming 20 mL of water was significantly different from that after consuming 150 or 240 mL of water. The viscosity after consuming 20 mL of water slowly decreased, but those after consuming 150 or 240 mL of water remained almost unchanged as a function of time.

In the Fed III condition, 240 mL was administered with the TD-TAB and TDF-1 formulations to maintain a similar viscosity in the stomach. However, the T/R of C_max_ and AUC were 0.635−0.847 and 0.929–1.074, respectively (Figure 4d). Based on the PK data after administering 20 mL (Fed I) or 240 mL (Fed III) water, the difference in the relative viscosity of meal contents in the stomach did not significantly affect modulating the low C_max_ of the TDF formulation. This result implies that relative viscosity in the stomach may not directly correlate with the behavior of the formulation, commonly known as delayed GET [26,27,28].

Under postprandial conditions, both dosage forms are inevitably influenced by the digestion and dilution behavior of the meal content in the stomach, which acts as a medium for drug dissolution. One important difference between the fasting and fed states is the duration for which the digestion medium passes through the pylorus, which shows a substantial difference in GET [58,59]. In the Fed I, Fed II, and Fed III conditions, the influence of drug particle size reduction (TDF-2) or the increased amount of water (240 mL) consumed with TDF-1 during the first meal intake (breakfast) did not significantly affect the drug behavior and pharmacokinetics of TDF. At this stage, we focused on the second meal intake time (lunch), which might influence the postprandial behavior of the TDF formulation.

In the Fed IV study, the second meal intake time was extended from 4 to 6 h, allowing the stomach to completely empty the meal contents after the first meal intake (breakfast). Surprisingly, the plasma concentration profiles of TDF-1 after increasing the second meal intake time were significantly different from those of other fed states (Figure 4e). Furthermore, the double-peak phenomenon was not observed. For the bioequivalence analysis in the Fed IV condition, the T/R of C_max_ and AUC were 0.851–0.998 and 0.958–1.045, respectively, giving bioequivalence and legal substitution between TD-TAB and TDF-1.

### 3.5. Mechanistic Understanding of the Importance of Second Meal Intake Time

In a CFD model study, large particles and solids move from the top to the bottom of the stomach in a fed state, whereas liquid and light particles move from the top of the stomach through terminal antrum contraction [33,34]. The particles are gradually broken down by gastrointestinal movements such as tonic contraction (TC) and antrum contraction (AC), and eventually progress through the digestive path for gastric emptying. At this stage, large solid particles are located towards the bottom of the stomach, resulting in physical stimulation and fast gastric emptying, owing to the influence of TAC. Over time, relatively heavier and denser solids move quickly through the pylorus, gradually decreasing the stomach viscosity.

According to previous in vivo studies on the disintegration of tablets, the disintegration time of immediate-release tablets in the stomach can generally be delayed by more than two times in the postprandial state compared to that in the fasting state [26,27,28], most likely due to the viscosity. Moreover, the disintegration start time of administered immediate-release tablets can be more than 10 min [28], and the influence of the wetting delay and gastric viscosity of coated tablets can be considered [27]. The TD-TAB formulation was likely to disintegrate in the lower part of the stomach shortly after settling by gravity, following administration. In contrast, TDF is disintegrated or dissolved by saliva and enters the stomach in a liquid state, then moves close to the surface, along with the liquid and light particles placed at the top of the stomach [33,34]. Drug dispersion and distribution from the dosage forms could begin depending on its gastric location. In this case, drug diffusion from TD-TAB began at the bottom of the stomach, whereas that from TDF formulation diffused out from the top of the stomach. 

The optimal tadalafil (TD)-loaded film, TDF-1, and TDF-2 were rapidly disintegrated and gave 40.8 ± 3.76 and 40.8 ± 5.84 s for TDF-1 and TDF-2, respectively. The dissolution rate was independent of the disintegration time of TDF formulations. Therefore, the TD from TDF was disintegrated in the oral cavity and TD was entered into the upper stomach, while TD from the conventional tablet was disintegrated into the lower region of the stomach. 

The biopharmaceutical factors affecting the drug’s passage through the stomach were mainly the viscosity and the resulting diffusion rate of the drug from dosage forms [36]. In previous studies, the average salivary flow rate when stimulated with a meal was 1.5–2 mL/min [60,61]. In this study, the average salivary flow rates ranged from 1.5 to 2 mL/min. Considering the known duration of meal intake, the amount of saliva secreted during meal intake was approximately 50 mL. The amount of inorganic and organic substances in the ingested food is negligible and less than approximately 1% of saliva [62]. Thus, a solution that considers only the pH-buffering function of saliva (sodium phosphate, pH 6.8) was used as a substitute for saliva [60]. This study did not account for the impact of chemical digestion, which provides numerous elements and functions and significant individual differences. In the fasting state, the amount of gastric juice is relatively small and has no viscosity [63,64,65]. To simulate the average gastric fluid and pH in the fasting and fed states, ground fat-simulated meal compositions were mixed with 50 mL sodium phosphate (pH 6.8) and 100 mL 0.01 N HCl (pH 2.0 ± 0.2) by varying the amount of water [64].

Owing to the difference in the location of the dosage forms, TD-TAB might pass more rapidly through the pylorus of the stomach from the lower region. In contrast, TDFs may experience a delay in passing through the lower pylorus after administration, owing to prolonged diffusion time. This also suggests that the delayed GET could be significantly influenced by the location of the beginning of diffusion. The increased viscosity due to guar gum content is determined by the homogenization time of the liquid in a study to mimic the mechanical action of gastrointestinal motility [36]. This indicates that dosage forms may have considerable time to diffuse in a high-viscosity gastric environment. These behaviors, together with first and second meal intake times, could critically impact the decreasing tendency of C_max_ and the potential delay of T_max_ in bioequivalence studies.

**Figure 6:**
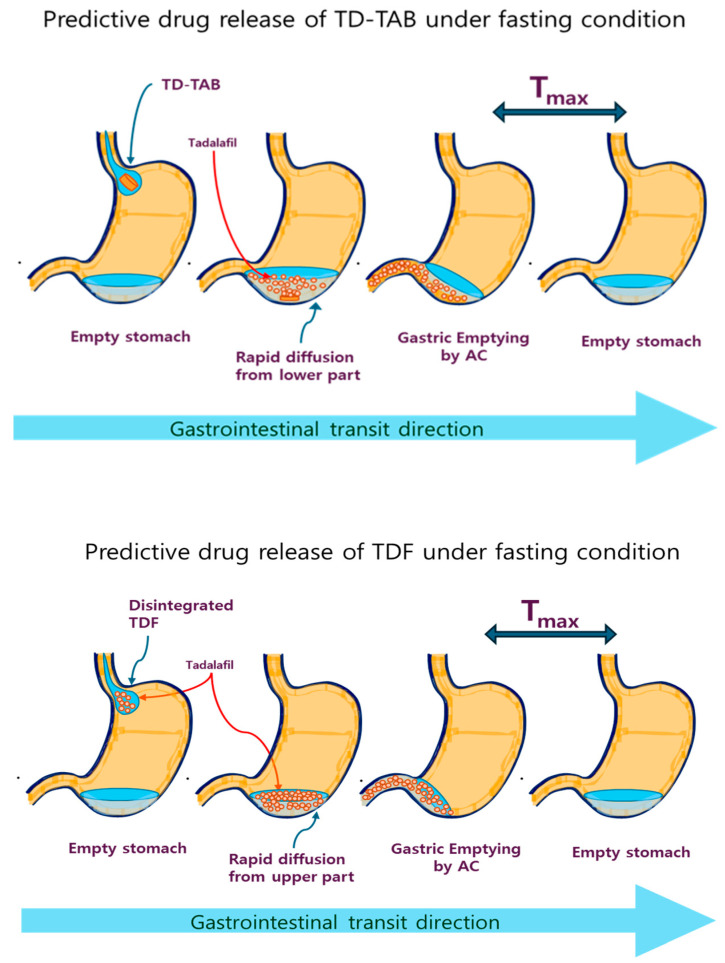
The predictive diffusion direction model of TD-TAB (**top**) and TDF-1 (**bottom**) in the stomach under a fasting state.

Based on these findings, the predictive diffusion direction model of TD-TAB and TDF in the stomach under the fasting state (no breakfast) and second meal intake (lunch at 4 h after dosing), or after first meal (breakfast) and second meal (lunch at 4 or 6 h after dosing), should be carefully understood to evaluate the PK and bioequivalence of TDF formulations. GET can vary among individuals in the fasting state but is typically completed within approximately 30 min [58]. Accordingly, both the TD-TAB and TDF formulations were likely to pass through the pylorus within this time in the fasting state, regardless of whether drug diffusion in the formulation was completed. Figure 6 shows the predictive diffusion direction model in the stomachs of TD-TAB and TDF-1 mice in the fasting state. As the entire dose (strength) of the drug passes through the pylorus in a short period (approximately 30 min), the T_max_ and corresponding C_max_ between the two formulations are more likely to be similar without interfering with the meal content, resulting in bioequivalence. Furthermore, no double-peak phenomenon is observed, yielding a T_max_.

**Figure 7:**
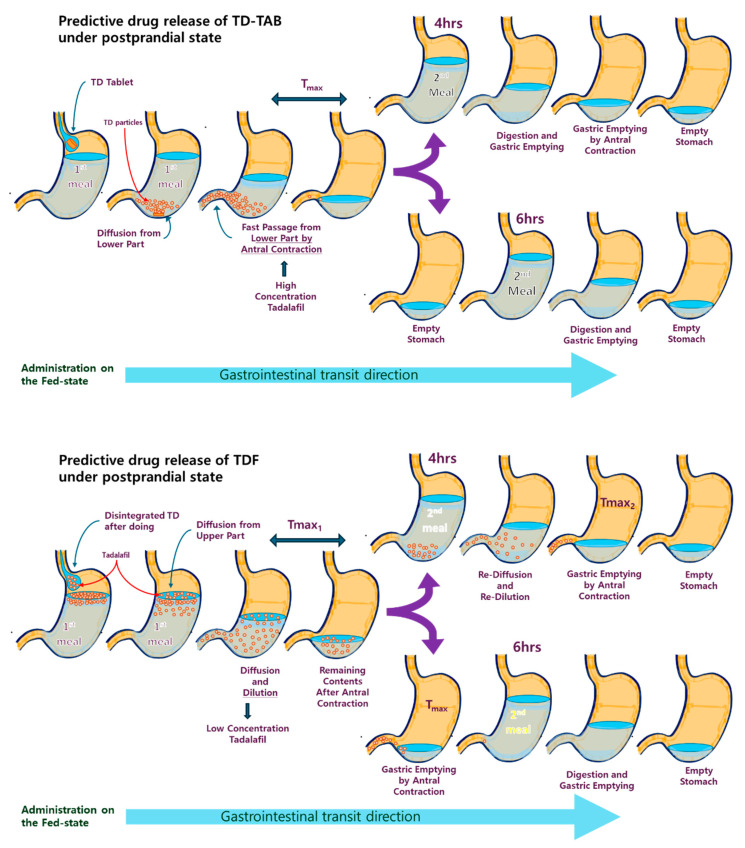
Predictive diffusion direction model in the stomach of TD-TAB (**top**) and TDF-1 (**bottom**) under fed state after the first meal intake (breakfast) before drug dosing, followed by the second meal intake (lunch), 4 h or 6 h after drug dosing.

In contrast, in the postprandial state, the speed of GET is significantly reduced because of differences in the size and viscosity of dosage forms, together with the ingested meal contents in the stomach and meal intake time [33,34]. This delay in the GET may prolong drug diffusion from the dosage form. Figure 7 shows the predictive diffusion direction model for TD-TAB and TDF-1 in the fed state. TD-TAB disintegrated in the lower stomach, and the diffusion rate of the drug notably decreased in the postprandial state. TD particles in the lower part of the stomach quickly pass through the pylorus because of their proximity to the pylorus and the antral contractile effect of the lower stomach [33,34].

In the case of the TDF formulation in the fed state with a second meal intake 4 h after dosing, the increase in C_max_ from TD-loaded TDF was inevitably delayed, since GE of the drug was possible only after diffusion from the upper layer of the stomach and homogenization of the stomach contents. In addition, the second meal intake 4 h after drug dosing was re-diluted with the drug and the remaining contents in the stomach, leading to a re-increase in blood drug concentration. Therefore, two C_max_ peaks were expected, as shown in Figure 3. This prediction was consistent with the occurrence of the double-peak phenomenon in the TDF formulations, as also observed in the Fed I, II, and III conditions. However, the effect of food on the bioavailability of TD-TAB was minimal. Furthermore, no double-peak phenomenon of TD-TAB was observed under fasting and fed conditions with first or second meal intake. Thus, the second meal intake time after TDF dosing was critical for increasing the C_max_ and delaying the T_max_.

To avoid any unwanted influence of meal contents in the stomach, sufficient time intervals between the first and second meal intake must be considered for sufficient GE of the drug to be delayed by TDF diffusion direction or location and the digestion time of stomach contents under postprandial conditions. The digestion time of food is 3–4 h [66]. However, the effect of meal interval time varies widely depending on environmental and individual differences; therefore, the delay in the second meal time was within an acceptable range [67,68].

In the bioequivalence guidelines under fed conditions, additional food intake is restricted to more than 4 h after oral administration, likely considering the typical digestion time. However, no specific time limit was set for the second meal intake, possibly to facilitate flexible bioequivalence studies on the optimal drug dosage and administration according to the indications. Although the second meal is commonly administered 4 h after oral dosing, the second meal intake time was increased to 6 h (Fed IV) after oral TDF-1 dosing to minimize the drug re-dilution effect caused by the second meal, which acted as an additional factor influencing the absorption process. Interestingly, C_max_ increased, but T_max_ reduced for TDF, giving bioequivalence with TD-TAB without showing the double-peak phenomenon. This indicated that the second meal intake time significantly affects the behavior of gastric fluid volume and gastric emptying of water and meal contents, contributing to drug dissolution and postprandial behaviors of diverse dosage forms [58], although generalizing the intersubject variability and bioequivalence of various dosage forms depending on BCS classification, drug type, or in vivo environment is difficult. If the mealtime interval exceeded 6 h, there was a high possibility of bioequivalence, as shown in the Fed IV condition, although mealtime intervals longer than 6 h were of low significance.

## 4. Conclusions

TD-TAB can be consumed with or without food. In the fasting state, no significant differences were observed between the TD-TAB and TDF groups. Furthermore, the drug particle size and meal viscosity did not significantly affect the bioequivalence of TD-TAB and TDF in the fed state. However, the second meal intake time, 4 or 6 h after oral dosing, significantly affected the pharmacokinetics and bioequivalence of TDF formulations in healthy human volunteers. In the Fed IV conditions, extending the interval of the second meal time from 4 to 6 h was important for the bioequivalence of TDF and TD-TAB, without showing a double-peak phenomenon. The predicted diffusion direction and drug release by digestion and gastric emptying in the stomach of TD-TAB and TDF by the varying second meal intake time was successfully simulated through CFD modeling. Owing to the unique characteristics of the TDF formulation and the considerable variability in individual meal timings, a flexible and broad range of second meal intake times beyond 4 h should be recommended for TDF formulations. The current findings, emphasizing the importance of second meal intake time, could provide an important guideline for further understanding PK and bioequivalence of orally disintegrating or dissolving formulations in postprandial conditions.

## Figures and Tables

**Table 1:** Dosing schedule of ODFs for investigating pharmacokinetics and bioequivalence in healthy human volunteers under fasting and fed states.

Study	TDF Formulation	Water Intake Volume (mL)	First Meal Intake Time (Breakfast)	Second Meal Intake Time (h) after Administration
Fasting	TDF-1	20	No	4
Fed I	TDF-1	20	Yes	4
Fed II	TDF-2	20	Yes	4
Fed III	TDF-1	240	Yes	4
Fed IV	TDF-1	20	Yes	6

**Table 2:** The visual appearance and % RSD of polymers with dispersion agents.

Polymer	Dispersion Agent	% RSD	Visual Appearance
HPMC (5 cp)	Polyethylene glycol 400	0.44	Surface crack, Surface roughness
	Polyoxyl 35 castor oil	2.79	Surface roughness, TD aggregation
	Polyethylene glycol 40 hydrogenated castor oil	3.08	TD aggregation
	Triethyl citrate	2.84	Non-homogenous layer
	Polyethylene oxide	3.83	Surface crack
	Polyvinylalcohol–Polyethylene glycol copolymer	3.82	Non-homogenous layer
HPC-SL	Polyethylene glycol 400	0.43	Very good
	Polyoxyl 35 castor oil	2.46	TD aggregation, Surface crack
	Polyethylene glycol 40 hydrogenated castor oil	2.52	Surface crack
	Triethyl citrate	3.11	Non-homogenous layer
	Polyethylene oxide	2.65	Surface roughness
	Polyvinylalcohol–Polyethylene glycol copolymer	2.72	Non-homogenous layer
Pullulan	Polyethylene glycol 400	0.31	Surface roughness
	Polyoxyl 35 castor oil	2.68	TD aggregation
	Polyethylene glycol 40 hydrogenated castor oil	2.54	Surface roughness
	Triethyl citrate	3.02	Non-homogenous layer
	Polyethylene oxide	3.04	TD aggregation, Surface roughness
	Polyvinylalcohol–Polyethylene glycol copolymer	2.99	Non-homogenous layer

RSD: Relative standard deviation, TD: tadalafil.

**Table 3:** Comparative pharmacokinetic parameters of TDF and reference TD-TAB (Cialis^®^) in healthy human volunteers under fasting and fed states.

Condition	Items	Variable				
		C_max_ (ng/mL)	T/R, 90% CI	AUC_t_ (h*ng/mL)	T/R, 90% CI	T_max_ (h)
Fasting (*n* = 37)	TD-TAB	268.2 ± 77.7	0.936–1.105	6399.8 ± 2447.9	1.012–1.153	2.6 ± 1.7
	TDF-1	276.3 ± 83.9		6871.8 ± 2234.5		3.2 ± 2.2
Fed I (*n* = 10)	TD-TAB	416.4 ± 81.0	0.610–0.798	6562.4 ± 1269.4	0.884–1.022	2.3 ± 1.0
	TDF-1	290.4 ± 81.0		6237.3 ± 1548.0		7.9 ± 3.2
Fed II (*n* = 12)	TD-TAB	326.8 ± 77.4	0.735–0.972	6549.6 ± 3034.0	0.894–1.137	2.6 ± 2.6
	TDF-2	276.2 ± 100.3		6602.3 ± 3028.6		5.7 ± 6.1
Fed III (*n* = 10)	TD-TAB	408.6 ± 153.6	0.635–0.847	7097.6 ± 2631.7	0.929–1.074	3.2 ± 1.5
	TDF-1	299.6 ± 102.3		7089.9 ± 2525.1		6.8 ± 3.2
Fed IV (*n* = 8)	TD-TAB	400.8 ± 61.2	0.851–0.998	6354.9 ± 1542.9	0.958–1.045	2.4 ± 2.1
	TDF-1	367.3 ± 59.2		6382.5 ± 1501.7		4.8 ± 1.7

## Data Availability

The original contributions presented in the study are included in the article. Further inquiries can be directed to the corresponding author.

## Supplementary Materials

The following supporting information can be downloaded at https://www.mdpi.com/article/10.3390/pharmaceutics16070915/s1, Figure S1: Chemical structure of tadalafil; Figure S2: Particle size analysis of TD with different distributions used in TDF-1 (D90:12.4 μm) and TDF-2 (D90: 7.8 μm).

## Abbreviations

AC	antral contraction
ACW	antral contraction wave
AUC	area under the curve
BCS	biopharmaceutics classification
BE	bioequivalence
CI	confidence interval
CFD	computational fluid dynamics
GE	gastric emptying
GET	gastric emptying time
HPC-SL	hydroxypropyl cellulose SL
HPMC	hydroxypropylmethyl cellulose
IVIVC	in-vitro in-vivo correlation
LOD	loss on drying
ODF	orodispersible film
PET	polyethylene terephthalate
PSD	particle size distribution
RSD	relative standard deviation
SEM	scanning electron microscopy
TAC	terminal antrum contraction
TC	tonic contraction
TD	tadalafil
TDF	tadalafil-loaded ODF
TD-TAB	tadalafil-loaded tablet
T/R	test sample/reference sample ratio
