# Supplementary material for: Effects of Postprandial Factors and Second Meal Intake Time on Bioequivalence Investigation of Tadalafil-Loaded Orodispersible Films in Human Volunteers
Source: Pharmaceutics. 2024 Jul 9;16(7):915. doi: 10.3390/pharmaceutics16070915 (PMC11280306; doi:10.3390/pharmaceutics16070915)
Supplement: Supplementary file 1 [file pharmaceutics-16-00915-s001.zip › pharmaceutics-3046499-supplementary.pdf]

Effects of Postprandial Factors and Second Meal Intake Time on Bioequivalence Investigation of Tadalafil-Loaded Orodispersible Films in Human Volunteers

Su-Jun Park [1,2], Myung-Chul Gil [3], Bong-Sang Lee [3], Minji Jung [4] and Beom-Jin Lee [1,5,*]

[1] Department of Pharmacy, College of Pharmacy, Ajou University, Suwon-si 16499, Republic of Korea; klevel2@hanmail.net-si
[2] CTCBIO Inc., Hwaseong-si 18576, Republic of Korea
[3] PLUTO Inc., Seongnam-si 13453, Republic of Korea; mcgil@pluto5.co.kr (M.-C.G.); keberos@pluto5.co.kr (B.-S.L.)
[4] Department of Urology, School of Medicine, Stanford University, Stanford, CA 94305, USA;
mjjung@stanford.edu
[5] Institute of Pharmaceutical Science and Technology, Ajou University, Suwon-si 16499, Republic of Korea

* Correspondence: bjl@ajou.ac.kr (Beom-Jin Lee), Tel.:+82-31-219-3442

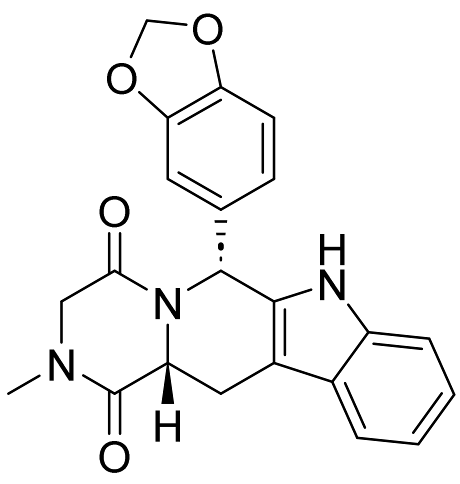

Figure S1. Chemical structure of tadalafil (TD).

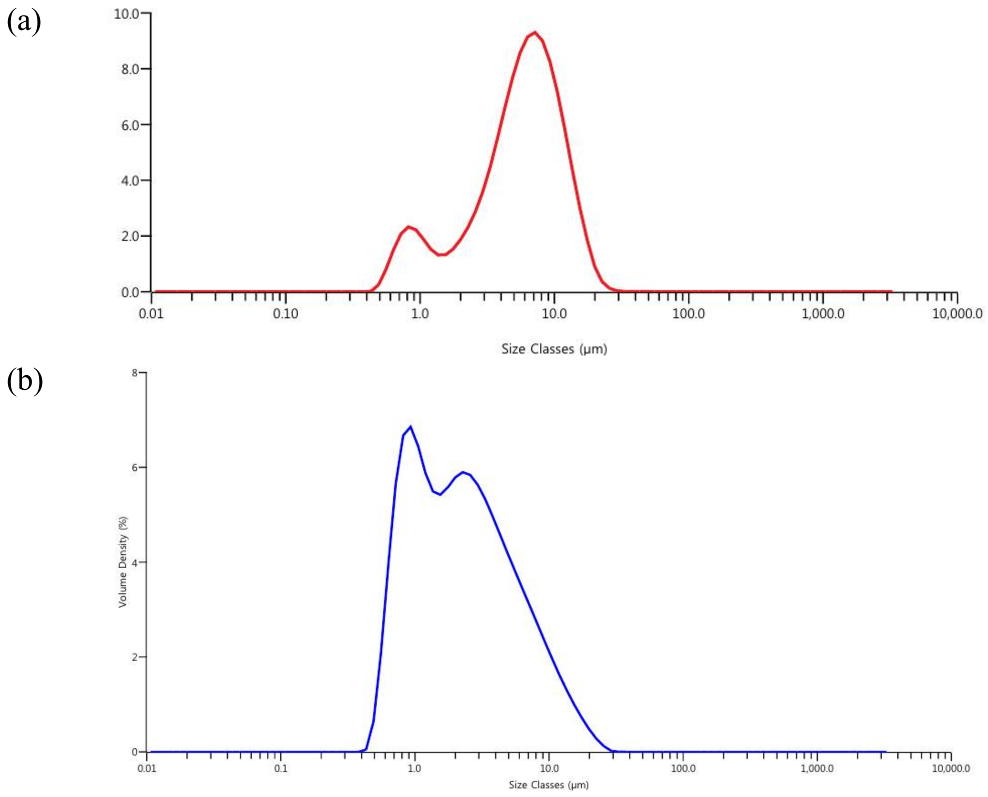

Figure S2. Particle size analysis of TD with different distributions used in TDF-1 (D90:12.4μm) and TDF-2 (D90: 7.8 μm).
